# *N*′-Benzyl­idene­furan-2-carbohydrazide

**DOI:** 10.1107/S1600536810021471

**Published:** 2010-06-18

**Authors:** Yu-Feng Li, Fang-Fang Jian

**Affiliations:** a Microscale Science Institute, Department of Chemistry and Chemical Engineering, Weifang University, Weifang 261061, People’s Republic of China; b Microscale Science Institute, Weifang University, Weifang 261061, People’s Republic of China

## Abstract

In the title compound, C_12_H_10_N_2_O_2_, the dihedral angle between the benzene ring and the furan ring is 24.6 (2)°. In the crystal, mol­ecules are linked by N—H⋯O hydrogen bonds, generating *C*(4) chains propagating in [010].

## Related literature

For background to Schiff bases as ligands, see: Polt *et al.* (2003 ▶). For a related structure, see: Jiang (2010 ▶).
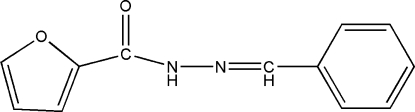


## Experimental

### 

#### Crystal data

C_12_H_10_N_2_O_2_
*M* *_r_* = 214.22
Orthorhombic, 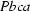

*a* = 11.628 (2) Å
*b* = 7.6638 (15) Å
*c* = 23.873 (5) Å
*V* = 2127.4 (7) Å^3^
*Z* = 8
Mo *K*α radiation
μ = 0.09 mm^−1^
*T* = 293 K
0.22 × 0.20 × 0.18 mm

#### Data collection

Bruker SMART CCD diffractometer
15748 measured reflections
1915 independent reflections
841 reflections with *I* > 2σ(*I*)
*R*_int_ = 0.180

#### Refinement

*R*[*F* ^2^ > 2σ(*F* ^2^)] = 0.069
*wR*(*F* ^2^) = 0.182
*S* = 0.87
1915 reflections
146 parameters
H-atom parameters constrained
Δρ_max_ = 0.48 e Å^−3^
Δρ_min_ = −0.35 e Å^−3^

### 

Data collection: *SMART* (Bruker, 1997 ▶); cell refinement: *SAINT* (Bruker, 1997 ▶); data reduction: *SAINT*; program(s) used to solve structure: *SHELXS97* (Sheldrick, 2008 ▶); program(s) used to refine structure: *SHELXL97* (Sheldrick, 2008 ▶); molecular graphics: *SHELXTL* (Sheldrick, 2008 ▶); software used to prepare material for publication: *SHELXTL*.

## Supplementary Material

Crystal structure: contains datablocks global, I. DOI: 10.1107/S1600536810021471/hb5485sup1.cif

Structure factors: contains datablocks I. DOI: 10.1107/S1600536810021471/hb5485Isup2.hkl

Additional supplementary materials:  crystallographic information; 3D view; checkCIF report

## Figures and Tables

**Table 1:** Hydrogen-bond geometry (Å, °)

*D*—H⋯*A*	*D*—H	H⋯*A*	*D*⋯*A*	*D*—H⋯*A*
N1—H1*A*⋯O2^i^	0.86	2.06	2.911 (4)	168

Symmetry code: (i) 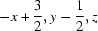.

**Fig. 1.**
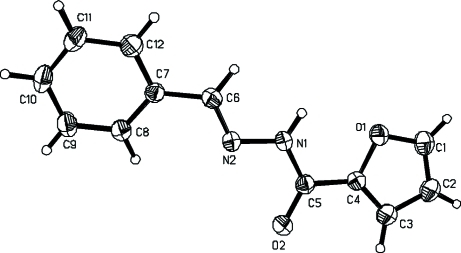
The structure of (I) showing 30% probability displacement ellipsoids.

## supplementary crystallographic information

### Comment

Metal complexes based on Schiff bases have attracted much attention because they can be utilized as effective ligands to form the compounds with optically active (Polt *et al.*, 2003). As part of our search for new Schiff base compounds we synthesized the title compound (I), and describe its structure here. The dihedral angle between the benzene ring and the furan ring is 24.6 (2)°. In the crystal lattice, the N—H···O hydrogen bonds which form chains stable the molecule structures.

Bond lengths and angles are comparable to those in a related material (Jiang, 2010).

### Experimental

A mixture of benzaldehyde (0.1 mol), and furan-2-carbohydrazide (0.1 mol) was stirred in refluxing ethanol (20 ml) for 2 h to afford the title compound (0.096 mol, yield 96%).  Colourless blocks of (I) were obtained by recrystallization from ethanol at room temperature.

### Refinement

H atoms were fixed geometrically and allowed to ride on their attached atoms, with C—H distances=0.97 Å, and with *U*_iso_=1.2–1.5*U*_eq_.

### Crystal data

**Table:**

C_12_H_10_N_2_O_2_	*F*(000) = 896
*M**_r_* = 214.22	*D*_x_ = 1.338 Mg m^−^^3^
Orthorhombic, *P**b**c**a*	Mo *K*α radiation, λ = 0.71073 Å
Hall symbol: -P 2ac 2ab	Cell parameters from 2542 reflections
*a* = 11.628 (2) Å	θ = 2.7–25.4°
*b* = 7.6638 (15) Å	µ = 0.09 mm^−^^1^
*c* = 23.873 (5) Å	*T* = 293 K
*V* = 2127.4 (7) Å^3^	Block, colorless
*Z* = 8	0.22 × 0.20 × 0.18 mm

### Data collection

**Table:**

Bruker SMART CCD diffractometer	841 reflections with *I* > 2σ(*I*)
Radiation source: fine-focus sealed tube	*R*_int_ = 0.180
graphite	θ_max_ = 25.3°, θ_min_ = 3.3°
phi and ω scans	*h* = −13→13
15748 measured reflections	*k* = −9→9
1915 independent reflections	*l* = −28→28

### Refinement

**Table:**

Refinement on *F*^2^	Secondary atom site location: difference Fourier map
Least-squares matrix: full	Hydrogen site location: inferred from neighbouring sites
*R*[*F*^2^ > 2σ(*F*^2^)] = 0.069	H-atom parameters constrained
*wR*(*F*^2^) = 0.182	*w* = 1/[σ^2^(*F*_o_^2^) + (0.0916*P*)^2^] where *P* = (*F*_o_^2^ + 2*F*_c_^2^)/3
*S* = 0.87	(Δ/σ)_max_ < 0.001
1915 reflections	Δρ_max_ = 0.48 e Å^−^^3^
146 parameters	Δρ_min_ = −0.35 e Å^−^^3^
0 restraints	Extinction correction: *SHELXL97* (Sheldrick, 2008), Fc^*^=kFc[1+0.001xFc^2^λ^3^/sin(2θ)]^-1/4^
Primary atom site location: structure-invariant direct methods	Extinction coefficient: 0.042 (4)

### Special details

**Table:**

Geometry. All e.s.d.'s (except the e.s.d. in the dihedral angle between two l.s. planes) are estimated using the full covariance matrix. The cell e.s.d.'s are taken into account individually in the estimation of e.s.d.'s in distances, angles and torsion angles; correlations between e.s.d.'s in cell parameters are only used when they are defined by crystal symmetry. An approximate (isotropic) treatment of cell e.s.d.'s is used for estimating e.s.d.'s involving l.s. planes.
Refinement. Refinement of *F*^2^ against ALL reflections. The weighted *R*-factor *wR* and goodness of fit *S* are based on *F*^2^, conventional *R*-factors *R* are based on *F*, with *F* set to zero for negative *F*^2^. The threshold expression of *F*^2^ > σ(*F*^2^) is used only for calculating *R*-factors(gt) *etc*. and is not relevant to the choice of reflections for refinement. *R*-factors based on *F*^2^ are statistically about twice as large as those based on *F*, and *R*- factors based on ALL data will be even larger.

### Fractional atomic coordinates and isotropic or equivalent isotropic displacement parameters (Å^2^)

**Table:**

	*x*	*y*	*z*	*U*_iso_*/*U*_eq_	
C7	0.6392 (3)	0.2814 (4)	0.22579 (15)	0.0427 (9)	
N2	0.7400 (2)	0.2911 (4)	0.13973 (12)	0.0444 (8)	
O2	0.89248 (19)	0.4222 (3)	0.06644 (10)	0.0525 (7)	
C6	0.6608 (3)	0.2226 (4)	0.16890 (15)	0.0464 (9)	
H6A	0.6161	0.1338	0.1537	0.056*	
N1	0.7595 (2)	0.2165 (4)	0.08817 (12)	0.0468 (8)	
H1A	0.7204	0.1273	0.0774	0.056*	
C11	0.5250 (3)	0.2748 (5)	0.30936 (18)	0.0614 (11)	
H11A	0.4599	0.2357	0.3282	0.074*	
C5	0.8408 (3)	0.2861 (4)	0.05515 (15)	0.0433 (9)	
O1	0.8127 (2)	0.0388 (3)	−0.00680 (11)	0.0624 (8)	
C12	0.5431 (3)	0.2243 (5)	0.25478 (17)	0.0565 (11)	
H12A	0.4905	0.1511	0.2372	0.068*	
C2	0.9298 (4)	0.0939 (6)	−0.07761 (19)	0.0717 (13)	
H2B	0.9697	0.0836	−0.1112	0.086*	
C8	0.7157 (3)	0.3882 (5)	0.25381 (16)	0.0499 (10)	
H8A	0.7816	0.4262	0.2354	0.060*	
C4	0.8663 (3)	0.1949 (4)	0.00321 (15)	0.0457 (9)	
C3	0.9374 (3)	0.2318 (5)	−0.03877 (18)	0.0619 (12)	
H4A	0.9835	0.3304	−0.0419	0.074*	
C10	0.6010 (4)	0.3816 (5)	0.33627 (18)	0.0617 (11)	
H10A	0.5884	0.4148	0.3732	0.074*	
C9	0.6968 (3)	0.4393 (5)	0.30781 (17)	0.0607 (11)	
H9A	0.7488	0.5134	0.3254	0.073*	
C1	0.8544 (4)	−0.0182 (6)	−0.05660 (18)	0.0746 (13)	
H1B	0.8329	−0.1222	−0.0737	0.090*	

### Atomic displacement parameters (Å^2^)

**Table:**

	*U*^11^	*U*^22^	*U*^33^	*U*^12^	*U*^13^	*U*^23^
C7	0.047 (2)	0.0397 (19)	0.042 (2)	0.0015 (16)	0.0025 (17)	0.0011 (17)
N2	0.0508 (17)	0.0434 (17)	0.0389 (19)	−0.0018 (14)	0.0016 (14)	−0.0050 (14)
O2	0.0574 (14)	0.0490 (15)	0.0512 (18)	−0.0076 (12)	0.0024 (13)	−0.0054 (12)
C6	0.052 (2)	0.0403 (19)	0.047 (2)	−0.0016 (17)	−0.0001 (19)	−0.0025 (17)
N1	0.0558 (18)	0.0463 (17)	0.0384 (19)	−0.0070 (14)	0.0023 (15)	−0.0081 (14)
C11	0.058 (2)	0.073 (3)	0.054 (3)	0.005 (2)	0.019 (2)	0.001 (2)
C5	0.0469 (19)	0.0411 (19)	0.042 (2)	0.0030 (17)	−0.0022 (18)	0.0021 (18)
O1	0.0769 (18)	0.0604 (17)	0.0499 (19)	−0.0157 (13)	0.0153 (14)	−0.0151 (13)
C12	0.051 (2)	0.060 (2)	0.059 (3)	−0.0034 (19)	0.007 (2)	−0.006 (2)
C2	0.085 (3)	0.080 (3)	0.051 (3)	−0.011 (2)	0.024 (2)	−0.006 (2)
C8	0.051 (2)	0.051 (2)	0.047 (3)	−0.0037 (18)	0.0013 (19)	−0.0039 (18)
C4	0.051 (2)	0.0434 (19)	0.043 (3)	−0.0017 (16)	−0.0007 (18)	−0.0012 (18)
C3	0.070 (3)	0.062 (3)	0.053 (3)	−0.010 (2)	0.012 (2)	−0.005 (2)
C10	0.080 (3)	0.068 (3)	0.038 (2)	0.016 (2)	0.007 (2)	−0.001 (2)
C9	0.073 (3)	0.062 (3)	0.048 (3)	0.002 (2)	−0.003 (2)	−0.010 (2)
C1	0.101 (3)	0.069 (3)	0.053 (3)	−0.007 (3)	0.019 (2)	−0.021 (2)

### Geometric parameters (Å, °)

**Table:**

C7—C8	1.382 (5)	O1—C4	1.370 (4)
C7—C12	1.385 (5)	C12—H12A	0.9300
C7—C6	1.453 (5)	C2—C1	1.327 (6)
N2—C6	1.268 (4)	C2—C3	1.408 (5)
N2—N1	1.376 (4)	C2—H2B	0.9300
O2—C5	1.234 (4)	C8—C9	1.365 (5)
C6—H6A	0.9300	C8—H8A	0.9300
N1—C5	1.342 (4)	C4—C3	1.329 (5)
N1—H1A	0.8600	C3—H4A	0.9300
C11—C10	1.366 (5)	C10—C9	1.377 (5)
C11—C12	1.376 (5)	C10—H10A	0.9300
C11—H11A	0.9300	C9—H9A	0.9300
C5—C4	1.454 (5)	C1—H1B	0.9300
O1—C1	1.357 (4)		
			
C8—C7—C12	117.7 (4)	C1—C2—H2B	126.9
C8—C7—C6	121.6 (3)	C3—C2—H2B	126.9
C12—C7—C6	120.6 (3)	C9—C8—C7	121.6 (4)
C6—N2—N1	116.0 (3)	C9—C8—H8A	119.2
N2—C6—C7	120.7 (3)	C7—C8—H8A	119.2
N2—C6—H6A	119.6	C3—C4—O1	109.7 (3)
C7—C6—H6A	119.6	C3—C4—C5	131.9 (3)
C5—N1—N2	118.4 (3)	O1—C4—C5	118.4 (3)
C5—N1—H1A	120.8	C4—C3—C2	107.4 (3)
N2—N1—H1A	120.8	C4—C3—H4A	126.3
C10—C11—C12	121.0 (4)	C2—C3—H4A	126.3
C10—C11—H11A	119.5	C11—C10—C9	119.0 (4)
C12—C11—H11A	119.5	C11—C10—H10A	120.5
O2—C5—N1	123.5 (3)	C9—C10—H10A	120.5
O2—C5—C4	119.5 (3)	C8—C9—C10	120.2 (4)
N1—C5—C4	117.0 (3)	C8—C9—H9A	119.9
C1—O1—C4	105.8 (3)	C10—C9—H9A	119.9
C11—C12—C7	120.6 (4)	C2—C1—O1	111.0 (4)
C11—C12—H12A	119.7	C2—C1—H1B	124.5
C7—C12—H12A	119.7	O1—C1—H1B	124.5
C1—C2—C3	106.1 (4)		

### Hydrogen-bond geometry (Å, °)

**Table:**

*D*—H···*A*	*D*—H	H···*A*	*D*···*A*	*D*—H···*A*
N1—H1A···O2^i^	0.86	2.06	2.911 (4)	168

Symmetry codes: (i) −*x*+3/2, *y*−1/2, *z*.
